# Exact score distribution computation for ontological similarity searches

**DOI:** 10.1186/1471-2105-12-441

**Published:** 2011-11-12

**Authors:** Marcel H Schulz, Sebastian Köhler, Sebastian Bauer, Peter N Robinson

**Affiliations:** 1 Max Planck Institute for Molecular Genetics, Ihnestr. 73, 14195 Berlin, Germany; 2 Ray and Stephanie Lane Center for Computational Biology, Carnegie Mellon University, 5000 Forbes Avenue, Pittsburgh, 15213 Pennsylvania, USA; 3 Institute for Medical Genetics and Human Genetics, Charité-Universitätsmedizin Berlin, Augustenburger Platz 1, 13353 Berlin, Germany; 4 Berlin-Brandenburg Center for Regenerative Therapies (BCRT), Charité-Universitätsmedizin Berlin, Augustenburger Platz 1, 13353 Berlin, Germany

## Abstract

**Background:**

Semantic similarity searches in ontologies are an important component of many bioinformatic algorithms, e.g., finding functionally related proteins with the Gene Ontology or phenotypically similar diseases with the Human Phenotype Ontology (HPO). We have recently shown that the performance of semantic similarity searches can be improved by ranking results according to the probability of obtaining a given score at random rather than by the scores themselves. However, to date, there are no algorithms for computing the exact distribution of semantic similarity scores, which is necessary for computing the exact *P*-value of a given score.

**Results:**

In this paper we consider the exact computation of score distributions for similarity searches in ontologies, and introduce a simple null hypothesis which can be used to compute a *P*-value for the statistical significance of similarity scores. We concentrate on measures based on Resnik's definition of ontological similarity. A new algorithm is proposed that collapses subgraphs of the ontology graph and thereby allows fast score distribution computation. The new algorithm is several orders of magnitude faster than the naive approach, as we demonstrate by computing score distributions for similarity searches in the HPO. It is shown that exact *P*-value calculation improves clinical diagnosis using the HPO compared to approaches based on sampling.

**Conclusions:**

The new algorithm enables for the first time exact *P*-value calculation via exact score distribution computation for ontology similarity searches. The approach is applicable to any ontology for which the annotation-propagation rule holds and can improve any bioinformatic method that makes only use of the raw similarity scores. The algorithm was implemented in Java, supports any ontology in OBO format, and is available for non-commercial and academic usage under: https://compbio.charite.de/svn/hpo/trunk/src/tools/significance/

## Background

Ontologies are knowledge representations using controlled vocabularies that are designed to help knowledge sharing and computer reasoning [1]. Many ontologies can be represented by directed acyclic graphs (DAGs), whereby the nodes of the DAG, which are also called *terms *of the ontology, are assigned to items in the domain and the edges between the nodes represent semantic *relations*. Ontologies are designed such that terms closer to the root are more general than their descendant terms. For the ontologies we consider in this paper, the *annotation-propagation rule *applies, that is, items are annotated to the most specific term possible but are assumed to be implicitly annotated to all ancestors of that term.

Examples for ontologies are the Foundational Model of Anatomy (FMA) ontology [2], the Sequence Ontology [3], the Cell Ontology [4], and the Chemical Entities of Biological Interest (ChEBI) ontology [5], which describe objects from the domains of anatomy, biological sequences, cells, and biologically relevant chemicals. In contrast, other ontologies are used to describe the attributes of the items of a domain. For instance, GO terms are used to annotate genes or proteins by describing the biological functions or characteristics to the proteins. The Mammalian Phenotype Ontology (MPO) [6] and the Human Phenotype Ontology (HPO) [7] describe the attributes of mammalian and human diseases. In this case, the domain object is a disease such as Marfan syndrome, whose attributes are the clinical features of the disease such as arachnodactyly and aortic dilatation. In other words, terms of phenotype ontologies such as the MPO and HPO can be conceived of as describing abnormal qualities (e.g. hypoplastic) of anatomical or biochemical entities [8].

Semantic similarity between any two terms within an ontology is based on the annotations to items in the domain and on the structure of the DAG. Different semantic similarity measures have been proposed [9,10] and the measures have been used in many different applications in computational biology. For example, different studies show that semantic similarity between proteins annotated with GO terms correlate with sequence similarity [11-13]. Other studies investigated the correlation of gene coexpression with semantic similarity using GO terms [14,15]. In addition, semantic similarity measures for GO terms have been used to predict protein subnuclear localization [16].

In another application we have implemented a semantic similarity search algorithm in the setting of medical diagnosis. A user enters HPO terms describing the clinical abnormalities observed in a patient and a ranked list of the best matching differential diagnoses is returned [17]. This kind of search can be performed using raw semantic similarity scores calculated using any of the semantic similarity measures [18,12,22]. However, among these different measures the node-based pairwise similarity measure defined by Resnik turned out to have the best performance in our previous study [17] and is therefore considered in this work.

The search is based on *q *attributes (HPO terms) that describe the phenotypic abnormalities seen in a patient for whom a diagnosis is being sought. For each of the entries of a database containing diseases annotated with HPO terms corresponding to their characteristic signs and symptoms, the best match between each of the *q *terms of the query with one of the terms annotating the disease is found and the average of the semantic similarity scores is determined. The diseases are then ranked according to these scores and returned to the user as suggestions for the *differential diagnosis*.

The distribution of scores that a domain object can achieve varies according to the number and specificity of the ontology terms used to annotate it. In a recent study by Wang et al. [23], it was discovered that many of the commonly used semantic similarity measures, including the ones used in this work, are biased towards domain objects that have more annotations. The effect was termed annotation bias. Applications that use the scores alone therefore tend to preferentially select items with higher numbers of annotations, which may lead to wrong conclusions [23].

Previously, we developed a statistical model to assign *P*-values to the resulting similarity scores on the basis of the probability of a random query obtaining at least as high a score in order to compensate for the fact that different domain objects may have a different number of annotations. Using extensive simulations, we showed that this approach outperformed searches based on the semantic similarity scores alone [17]. A disadvantage of that procedure was the fact that extensive simulations using randomized queries were necessary in order to estimate the true distribution of the semantic similarity scores, which is needed in order to calculate a *P*-value for any given similarity score.

In this paper, we describe an algorithm to collapse a DAG representing an ontology into connected components of nodes corresponding to terms that make identical contributions to the semantic similarity score. The new algorithm reduces the amount of computational time needed to calculate the score distribution (and thereby *P*-values) by many orders of magnitude compared to a naive calculation. A preliminary description of the algorithm was presented in a conference paper [24]. Here, we validate the algorithm by comparing to sampling based approaches and show using simulations that the application of the exact *P-*value outperforms sampling based approaches in the context of clinical diagnostics with the HPO.

## Methods

### Notation

We consider an ontology *O *composed of a set of *terms *that are linked via an *is-a *or *part-of *relationship. The ontology *O *can then be represented by a DAG G=(V,E), where every term is a node in *V *and every link is a directed edge in *E*. A directed edge going from node *n*_1 _to *n*_2 _is denoted *e*_1,2 _and we refer to *n*_2 _as the *parent *of *n*_1_. An *item i *is defined as an abstract entity to which terms of the ontology are annotated. Let *Anc*(*n*) be defined as the ancestors of *n*, i.e., the nodes that are found on all paths from node *n *to the root of G, including *n*. We note that the annotation-propagation rule states that if an item is explicitly annotated to a term *n*, it is implicitly annotated to *Anc*(*n*). In order to describe the implicit annotations we define $T^{IMPL}$. Let T be the set of terms that has been explicitly annotated to item *i*, then $T^{IMPL}=\cup_{n\in T}Anc\left(n\right)$, namely all terms that are annotated to item *i *and all their ancestors in G. Let the set of common ancestors of two nodes *n*_1 _and *n*_2 _be defined as *ComAnc*(*n*_1_, *n*_2_) = *Anc*(*n*_1_) ⋂ *Anc*(*n*_2_). Let *Desc*(*n*) be the set of descendant nodes of *n*, again including *n*. Note that in this notation descendant nodes are considered only once, even if there are multiple paths leading to them.

### Multisets

In what follows we need to compute the similarity also between a *multiset *and a set of terms. The concept of multisets [25] is a generalization of the concept of sets. In contrast to sets, in which elements can only have a single membership, the elements of multisets may appear more than once.

Formally, a multiset *M *is a set of pairs, *M *= {(*s*_1_, *m*_1_),..., (*s*_*d*_, *m*_*d*_)}, in which *s*_*i *_∈ *U *= {*s*_1_,..., *s*_*d*_} are the elements of the *underlying set U*. Furthermore, *m*_*i *_defines the *multiplicity *of *s*_*i *_in the multiset. The sum of the multiplicities of *M *is called the *multiset cardinality *of *M*, denoted |*M*|. Only multiplicities in the domain of positive integers are considered, i.e., *m*_*i *_∈ ℕ^+^. We define a *multi subset *relation between multiset *N *and multiset *M*, denoted *as N *⊆ *M*, as a generalization of the subset relation between two sets:

N⊆M⇔∀(s,n)∈N:∃m≥n:(s,m)∈M.

The *multiset coefficient *$M\left(n,q\right)=\left(\begin{array}{c} n+q-1\\ q \end{array}\right)$ denotes the number of distinct multisets of cardinality *q*, with elements taken from a finite set of cardinality *n*. It describes how many ways there are to choose *q *elements from a set of *n *elements if repetitions are allowed.

### Similarity measures

We will concentrate in this work on the class of similarity measures that are based on the information content (*IC*) of a node:

(1) IC(n)=-logp(n),

where *p*(*n*) denotes the frequency among all items in the domain of annotations to *n*, which implicitly contains all annotations of descendants of *n *due to the annotation-propagation rule. The information content is a nondecreasing function on the nodes of G as we descend in the hierarchy and is therefore *monotonic*. The similarity between two nodes was defined by Resnik as the maximum information content among all common ancestors [19]:

(2) $$sim\left(n_{1},n_{2}\right)=max\left\{IC\left(a\right)|a\in ComAnc\left(n_{1},n_{2}\right)\right\}.$$

Equation (2) provides a definition for the similarity between two terms. Other popular pairwise measures that additionally incorporate the *IC *of the query terms, for example [20,21], are not considered here (see Discussion).

One can extend this concept to define a similarity between two domain objects that are each annotated by multiple ontology terms by taking the average of the best pairwise similarities for all terms [11]:

(3) $$sim^{avg}\left(T_{1},T_{2}\right)=\frac{1}{\left|T_{1}\right|}\underset{n_{1}\in T_{1}}{\sum }\underset{n_{2}\in T_{2}}{\max}sim\left(n_{1},n_{2}\right).$$

Note that Eq. (3) is not symmetric [12], i.e., it is not necessarily true that $sim^{avg}\left(T_{1},T_{2}\right)=sim^{avg}\left(T_{2},T_{1}\right)$. We point out that in other works average often refers to a symmetric definition. Using the nomenclature of Pesquita *et al*. [9], Eq. (3) may be referred to as asymmetric best-match average, here average for short.

Instead of taking the average the maximum similarity between a term annotating one of the domain objects and a term annotating the other domain object can be used to define the following symmetric measure:

(4) $$sim^{max}\left(T_{1},T_{2}\right)=\underset{n_{1}\in T_{1},n_{2}\in T_{2}}{\max}sim\left(n_{1},n_{2}\right).$$

**Figure 1:**
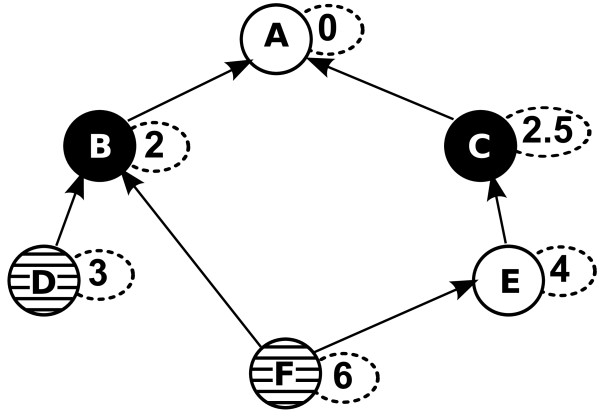
**Example Computation of *sim*^*avg*^**. Computation of *sim*^*avg *^on a DAG with six nodes. The target set is T={B,C} (black nodes) and the query set Q={D,F} (nodes with horizontal lines). The IC value of a node is shown in a small, dashed, attached oval. The most similar terms for *D *and *F *are *B *and *C *respectively, because *IC*(*B*) > *IC*(*A*) and *IC*(*C*) > *IC*(*B*). Therefore, $sim^{avg}\left(Q,T\right)=\frac{sim\left(D,B\right)+sim\left(F,C\right)}{2}=\frac{IC\left(B\right)+IC\left(C\right)}{2}=\frac{2+2.5}{2}$. Note that only terms involving nodes in $T^{IMPL}=\left\{A,B,C\right\}$ were considered in the calculation.

Equation (4) can be considered a simplified case of Eq. (3) because instead of averaging over all best-pairwise terms for each $n_{1}\in T_{1}$ compared to $n_{2}\in T_{2}$ only the highest similarity of all possible pairs is retained. Therefore, we will show the algorithm applied to Eq. (3) and sketch the changes for Eq. (4) later. One can use equation (3) or (4) to define a similarity between a set of query terms Q, i.e., $T_{1}=Q$ and an object in a database. Then, Q can represent any set of terms from the ontology *O *whereas $T_{2}$ refers to database objects (such as diseases annotated to HPO terms). As we are using this setup for the similarity queries we will omit the index and refer to $T_{2}$ as the target set T. See Figure 1 for an example computation of *sim*^*avg*^.

Because we later make use of scores derived at the maximization step in Eq. (3) we define:

(5) $$sim\left(n_{1},T\right)=\underset{n_{2}\in T}{\max}sim\left(n_{1},n_{2}\right),$$

to be the *target set similarity score *of *n*_1 _against a target set T. To avoid confusion we will denote scores of the score distribution of *sim*^*avg *^by *S *and target set similarity scores sim(n,T) by *s*.

### Definition of statistical significance for semantic similarity scores

In this paper we will present methods for analytically calculating the probability distribution of similarity scores for comparisons between a query set Q with *q *terms against an item that has been annotated with a *target set *T of nodes. For example, if a clinician chooses a set Q of HPO terms describing abnormalities seen in a patient and uses Eq. (3) to calculate an observed score *S*_*obs *_to a disease that has been annotated with terms of the HPO, we would like to know the probability of a randomly chosen set of *q *nodes achieving a score of *S*_*obs *_or greater. In this case, each disease in the database represents a *target set *(for instance, there are currently over 5000 diseases in the clinical database used by the Phenomizer at the HPO Web site).

In other words, our methods will be used to calculate a *P-*value for the null hypothesis that a similarity score of *S*_*obs *_or greater for a set of *q *query terms Q and a target set T has been observed by chance. We take all queries to be equally likely and define the *P-*value to be the proportion of queries having a score of at least *S*_*obs*_:

(6) $$P_{q,T}^{sim}\left(S\geq S_{obs}\right)=\frac{\left|\left\{Q|sim\left(Q,T\right)\geq S_{obs},Q=\left\{n_{1},...,n_{q}\right\}\subseteq V\right\}\right|}{\left(\underset{q}{|V |}\right)}.$$

In this definition all nodes of *V *can be part of a query, even if one node is an ancestor of the other. Note that the number of distinct scores for the complete score distribution of $P_{q,T}^{sim}$ is dependent on q,T, and the similarity measure.

### Simulation of patients for clinical diagnosis

Similar to our previous work [17], we use simulations to compare different approaches. Using 1701 OMIM diseases currently annotated with 2-5 HPO terms in the *Phenotypic abnormality *subontology, we generated artificial queries by *(i) *taking all terms annotated to the disease with no noise or imprecision as the query (NONE), *(ii) *randomly exchanging one term if *q *= 3 or *q *= 4 and two terms if *q *= 5 (NOISE), *(iii) *with probability 0.5 exchange a term with one of its parent terms if possible, (IMPRECISION), or *(iv) *using first IMPRECISION then NOISE.

For each of the 1701 OMIM diseases we generate the query as described above and rank all diseases using one of the measures (Score, *P*-value sampled 10^3^, 10^4^, or 10^5 ^times, and *P*-value exact). We then calculate the rank of the disease from which the query was generated. In case of ties we take the average rank (e.g. if four diseases rank first with the same value, all four get rank 2.5). Note that for the rankings using *P*-values (sampled or exact) we ranked first by *P*-values and then by score.

## Results

### A naive algorithm: exhaustive computation of score distributions

We represent the score distribution as $SD=\left\{\left(S_{1},F_{1}\right),\ldots ,\left(S_{k},F_{k}\right)\right\}$. Every pair $\left(S_{i},F_{i}\right)\in SD$ contains a unique score *S*_*i *_and a count *F*_*i *_that defines its frequency within the distribution.

A naive approach to calculating the complete score distribution is to determine the similarity of each possible term combination *Q *⊆ *V *of size *q *with the fixed target set T. The complete procedure is outlined in Algorithm 1. It requires two basic operations that are applied to the set SD. The first operation called *getScorePair *returns the pair that represents the given score or *nil *in case the entry does not exist. The second operation denoted *putScorePair *puts the given pair into the set SD, overwriting any previously added pair with the same score. For further analyses we assume that both operations have constant running time.

   **Input**: *V*, *q*, T

   **Output**: Score distribution $SD=\left\{\left(S_{1},F_{1}\right),\ldots ,\left(S_{k},F_{k}\right)\right\}$

1 SD=∅

2 **foreach ***Q *= {*n*_1_, *n*_2_,..., *n*_*q*_} ⊆ *V ***do**

3   $S_{new}\leftarrow sim^{avg}\left(Q,T\right)$

4   $\left(S,F\right)\leftarrow getScorePair\left(SD,S_{new}\right)$

5   **if **(*S*, *F*) ≠ *nil ***then**

6      $putScorePair\left(SD,\left(S_{new},F+1\right)\right)$

7   **else**

8      $putScorePair\left(SD,\left(S_{new},1\right)\right)$

9 **return **SD

**Algorithm 1**: Naive score distribution computation for *sim*^*avg*^

As the number of possible term combinations is $\left({}_{q}^{\left|V\right|}\right)$ and each similarity computation (line 3) costs O(q⋅|T|) operations for Eq. (3) Algorithm 1 runs in $O\left(\left|V{|}^{q}\cdot q\cdot |T\right|\right)$ time. A typical size of |*V*| = 10000 as for the HPO demonstrates that the naive approach is impractical for values *q *> 2. The naive approach neglects the relationships of the nodes in G and T. We will exploit these relationships in the next section and group nodes in G according to their contribution to the score distribution computation.

### A faster algorithm: exploiting redundant computations

Recall that all terms from the target set T are contained in $T^{IMPL}$. We will prove now that only the *IC *values of nodes in $T^{IMPL}$ are relevant for the score distribution computation.

**Lemma 1**. *Given a DAG *G=(V,E)*and a target set *$T=\left\{n_{1},\ldots ,n_{k}\right\}\subseteq V$, *all scores in the score distribution of the similarity measure of Eq. (*3*) are derived from *IC *values of the nodes in *$T^{IMPL}$.

*Proof*. Computing the complete score distribution involves repeatedly evaluating $sim^{avg}\left(Q,T\right)$ in Alg. 1 using equation (3). The first step for the computation of Eq. (3) is to maximize *sim*(*n*_1_,*n*_2_) for each node $n_{1}\in Q$ compared to nodes $n_{2}\in T$. The maximum *IC *value for *sim*(*n*_1_, *n*_2_) must be taken from a node in $T^{IMPL}$, because by definition $Anc\left(n_{2}\right)\subseteq T^{IMPL}$.

**Figure 2:**
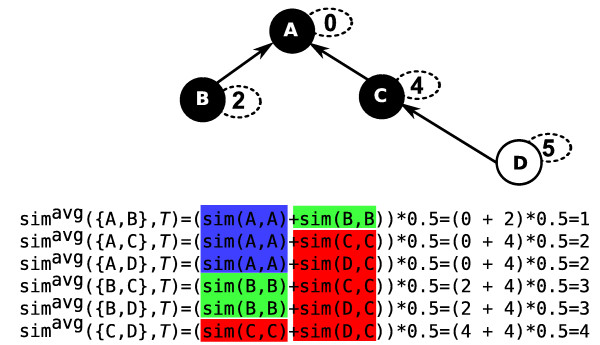
**Redundancy in Naive Score Distribution Computation with *sim*^*avg *^for Queries of Size Two**. Computation of the score distribution for *sim*^*avg *^on a DAG G with four nodes for all possible queries of size two. The target set T={A,B,C} is shown as black nodes. Note that $T=T^{IMPL}$ here. The *IC *value for nodes is shown in a small dashed oval. All computations of Eq. (5) that result in the same target similarity score are colored in blue, green, and red for the target set similarity scores 0, 2, and 4, respectively.

Lemma 1 implies that the computations in the naive algorithm, which enumerates all nodes in *V*, are highly redundant as the size of $T^{IMPL}$ is an upper bound on the number of different target set similarities encountered during score distribution computation. Figure 2 shows the contribution of all possible queries of size *q *= 2 for an example ontology. For instance, whenever node *C *or *D *are part of a query the target set similarity score obtained from Eq. (5) is *IC*(*C*) = 4, highlighted in red in Figure 2, and used for computing $sim^{avg}\left(Q,T\right)$.

Therefore, instead of enumerating over the nodes in *V*, we will first group nodes that have the same target set similarity score *s *in the maximization step in Eq. (3). Denote all nodes *n *∈ V that have the same target set similarity score *s *for a given target set T as *N*_*s*_:

(7) $$N_{s}=\left\{n|n\in V,sim\left(n,T\right)=s\right\}.$$

**Example 1**. It can be seen in Figure 2 that *N*_0 _= {*A*}, *N*_2 _= {*B*}, and *N*_4 _= {*C*, *D*} for G with T={A,B,C}.

Observe that two nodes $n_{i},n_{j}\in T^{IMPL},n_{i}\neq n_{j}$, belong to the same set *N*_*s*_, if *IC*(*n*_*i*_) = *IC*(*n*_*j*_). This observation will be essential when we devise an algorithm for computing *N*_*s*_.

The intuition behind the fast computation is that instead of selecting combinations of all nodes of *V *and constructing the score distribution one by one, we focus on the combinations of different target set similarity scores *s *and use their frequency |*N*_*s*_| to avoid redundant enumeration. For any T the set U of distinct target set similarity scores is defined as:

(8) $$U=\left\{IC\left(n\right)|n\in T^{IMPL}\right\}.$$

Instead of considering sets of nodes in *V *we will now consider multisets *M*^*q *^of target set similarity scores in U, where |*M*^*q*^| = *q*. In order to do that we define as M the multiset induced by all target similarity scores *s *and their corresponding multiplicities *m*, that is,

(9) $$M=\left\{\left(s_{1},m_{1}\right),\ldots ,\left(s_{d},m_{d}\right)|s_{i}\in U,m_{i}=|N_{si}|\right\}.$$

Then $M_{all}^{q}$ represents the set of all multi subsets of M that have multiset cardinality *q*, i.e.,

(10) $$M_{all}^{q}=\left\{M^{q}|M^{q}\subseteq M,|M^{q}|=q\right\}.$$

The value of *sim*^*avg *^computed for a particular *M*^*q *^is the same for all query sets of nodes that correspond to *M*^*q *^(see Figure 2, Example 2). Therefore, if we can calculate the number of such sets as well as the score corresponding to each multiset *M*^*q *^of target set similarity scores in U, we can determine the distribution of similarity scores *sim*^*avg *^for all possible queries of any given size *q*.

Denote the similarity for a multiset *M*^*q *^as:

(11) $$sim^{avg}\left(M^{q}\right)=\frac{1}{q}\underset{\left(s,m\right)\in M^{q}}{\sum }m\cdot s.$$

The number of ways of drawing m nodes from a component of size |*N*_*s*_| can be calculated using the binomial coefficient. The total number of combinations is then the product of all binomial coefficients, denoted as the *multiset frequency *for a multiset *M*^*q*^:

(12) $$freq\left(M^{q}\right)=\underset{\left(s,m\right)\in M^{q}}{\prod }\left(\begin{array}{c} \left|N_{s}\right|\\ m \end{array}\right).$$

**Example 2**. In total there are 2 query sets with $sim^{avg}\left(Q,T\right)=2$ for the DAG in Figure 2, namely {*A*, C}, {*A, D*}. After preprocessing, we obtain N_0 _= {A}, *N*_2 _= {*B*}, and *N*_4 _= {*C, D*} (Example 1). Alg. 2 enumerates all valid multisets of cardinality 2 for the sets *N*_*s *_considering their size |N_s _|. The only way of attaining an average score of 2 is to select one node out of *N*_0 _and *N*_4_, represented by the multiset *M*^2 ^= {(0,1), (4,1)} for which *sim*^*avg*^(*M*^2^) = 2. The multiset frequency of *M*^2 ^gives the same result as shown in Figure 2, $freq\left(M^{2}\right)=\left(\begin{array}{c} \left|N_{0}\right|\\ 1 \end{array}\right)\cdot \left(\begin{array}{c} \left|N_{4}\right|\\ 1 \end{array}\right)=1\cdot 2=2$. Instead of iterating over two sets we consider one multiset.

**Theorem 1**. *Let *$SD=\left\{\left(S_{1},F_{1}\right),\ldots ,\left(S_{k},F_{k}\right)\right\}$*be the score distribution computed with sim*^*avg *^*for an ontology DAG *G=(V,E), *target set *T⊆V*and query size q. The frequency F with which any given score S occurs amongst all possible queries of size q is then*:

(13) $$F=\underset{M^{q}\in M_{all}^{q},\;sim^{avg}\left(M^{q}\right)=S}{\sum }freq\left(M^{q}\right).$$

A proof of Theorem 1 is provided in Appendix A and a faster algorithm based on Theorem 1 is shown in Alg. 2. We enumerate all distinct multisets of $M_{all}^{q}$ and add their frequency to the score distribution SD, instead of iterating over all sets of size *q *in Alg. 1, thereby reducing the number of operations. In order to apply the algorithm to score distribution computation for *sim*^*max*^, line 3 of Alg. 2 needs to be replaced. Instead of computing the average of all scores in the multiset, the maximum among them is assigned to *S*_*new*_.

### Preprocessing of the DAG for faster computation

So far we have neglected how we can compute the values $|N_{s}|,s\in U$ but we will introduce an efficient algorithm in this section. We denote the algorithm as preprocessing because computation of |*N*_*s*_| is independent of *q*. The preprocessing will divide the original graph into a set of connected components from which the |*N*_*s*_| values can be deduced.

   **Input**: $M_{all}^{q}$

   **Output**: Score distribution $SD=\left\{\left(S_{1},F_{1}\right),\ldots ,\left(S_{k},F_{k}\right)\right\}$

1 SD=∅

2 **foreach ***multiset *$M^{q}\in M_{all}^{q}$**do**

3   *S*_*new *_← *sim*^*avg*^(*M*^*q*^)

4   $\left(S,F\right)\leftarrow getScorePair\left(SD,S_{new}\right)$

5   **if **(*S*,*F*) ≠ *nil ***then**

6      $putScorePair\left(SD,\left(S_{new},F+freq\left(M^{q}\right)\right)\right)$

7   **else**

8      $putScorePair\left(SD,\left(S_{new},freq\left(M^{q}\right)\right)\right)$

9 **return **SD

**Algorithm 2**: Faster score distribution computation for *sim*^*avg*^

First, we invert the direction of all edges in *E *such that the edges are directed from the root towards the leaves of the DAG, and introduce edge weights *w*_*i,j *_to the edges of G. Let

(14) $$w_{i,j}=\left\{\begin{array}{cc} IC\left(n_{i}\right), & \text{if}\;n_{i}\in T^{IMPL}\\ \max\left\{w_{h,i}|e_{h,i}\in E\right\} & \text{otherwise} \end{array}.\right)$$

The edge weights are defined in a recursive manner. First, all weights of edges emerging from nodes in $T^{IMPL}$ are set. Then the maximum edge weight of all incoming edges for each node not in $T^{IMPL}$ are propagated to all outgoing edges of the node, and as such propagated throughout the graph. Computing the edge weights is efficiently done after the nodes of G have been sorted in topological order, see Alg. 3. We now iterate across all nodes *n*_*i *_∈ *V*. For each node $n_{i}\in V,n_{i}\notin T^{IMPL}$, there is at least one path that leads to the node $n_{j}=\underset{n_{k}\in T}{\mathrm{argmax}}sim\left(n_{i},n_{k}\right)$. If a node has multiple parents, then by construction of the edge weights, an edge with a maximum weight will be a member of a path to *n*_*j*_. We therefore remove all other incoming edges. If there are multiple incoming edges with an identical, maximum edge weight, one of them can be chosen arbitrarily and the others are removed (Alg. 3, lines 7-9). We now iterate over all remaining edges *e*_*i,j *_and remove all edges for which $n_{i},n_{j}\in T^{IMPL}$ holds (Alg. 3, lines 10-12). Note that exactly $\left|T^{IMPL}\right|$ many connected components *C*_*i *_one for each $n_{i}\in T^{IMPL}$ remain.

For all pairs of connected components such that *IC*(*n*_*i*_) = *IC*(*n*_*j*_) for $n_{i},n_{j}\in T^{IMPL},n_{i}\neq n_{j}$, the connected components *C*_*i *_and *C*_*j *_are merged to arrive at the desired sets $N_{s},s\in U$ (Alg. 3, lines 13-16).

**Figure 3:**
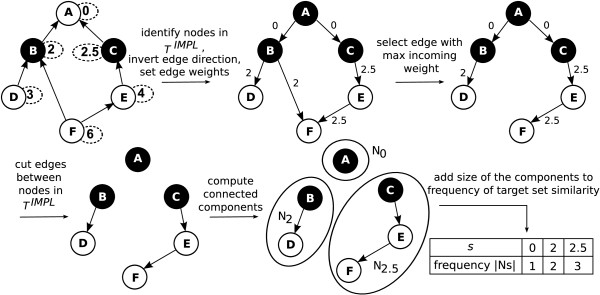
**Overview of the Algorithm for Preprocessing**. The general steps of Alg. 3 are shown on the DAG and T of Figure 1. Nodes in $T^{IMPL}$ are colored in black. The *IC *value of a node is depicted in a dashed oval.

All these steps are summarized in Alg. 3 and Figure 3.

**Theorem 2**. *Given a DAG *G=(V,E)*and a target set *$T=\left\{n_{1},\ldots ,n_{k}\right\}\subseteq V$*the score distribution of Eq. (3) is computed by Alg*. 2 *and Alg*. 3 *in *$O\left(|E|+|V|+M\left(|T^{IMPL}|,q\right)\right)$*time and space*.

*Proof*. The preprocessing of the *DAG *in Alg. 3 involves inverting edges, topological ordering of *V*,

   **Input**: *V*, $T^{IMPL}$

   **Output**: node sets with identical target similarity score, i.e., *N*_*s*_

1 **for ***n*_*i *_∈ *V in topological order ***do**

2   **for ***j in e*_*i,j *_∈ *E ***do**                                                                  /* Set weights */

3      **if **$n_{i}\in T^{IMPL}$**then**

4         *w*_*i,j *_← *IC*(*n*_*i*_)

5      **else**

6         *w*_*i*,*j *_← max{*w*_*h*,*I *_|e_*h*,*i *_∈ *E*}

7 **for ***n*_*i *_∈ *V *\ *root ***do**

8      choose *e*_*h*,*i *_∈ *E *s.t. |*w*_*h*,*i *_≥ *w*_*h',i *_for all edges *e*_*h',i*_∈ *E*

9      remove all incoming edges of *n*_*i *_except *e*_*h*,*i*_

10 **for ***e*_*i*,*j *_∈ *E ***do**                                                      /* Connected components*C*_i _*/

11      **if **$n_{i},n_{j}\in T^{IMPL}$**then**

12         remove *e*_*i*,*j *_from *E*

13 **for **$s\in \left\{IC\left(n_{i}\right)|n_{i}\in T^{IMPL}\right\}$**do**                              /* Merging */

14      $N_{s}=\emptyset$

15      **foreach **$n_{i}\in T^{IMPL}$**do**

16         *N*_*s *_← *N*_*s *_∪ *C*_*i*_

17 **return ***N*_*s*_

**Algorithm 3**: Graph preprocessing for faster computation

introducing edge weights to *E*, removing edges in *E*, and computing the connected components of G. This can be done with depth-first search (DFS) traversals of G with to a worst-case performance of O(|E|+|V|) time and space.

Algorithm 2 runs in $O\left(M\left(|T^{IMPL}|,q\right)\right)$ time and space. The outer *foreach *loop runs over all distinct multisets with cardinality *q*. The multiset coefficient $M\left(|T^{IMPL}|,q\right)$ provides an upper bound for the number of these multisets. In each iteration the computation of the similarity score (line 3) and the multiset frequency, *freq*(*M*^*q*^), have constant cost assuming a preprocessed lookup table for binomial coefficients and if common partial *sim*^*avg *^values are stored between the iterations, avoiding recomputation for similar multisets. In total, Alg. 2 and Alg. 3 run in $O\left(|E|+|V|+M\left(|T^{IMPL}|,q\right)\right)$ time and space.

The theorem concludes the improvement to the naive algorithm, for example on average $\left|T^{IMPL}\right|\sim 38$ for all diseases currently annotated with terms of the HPO, which currently has approximately 10000 terms and 13000 relations. For instance, for a query with 5 terms, the naive algorithm would thus run in time proportional to 10000^5 ^· 5 · 38 = 1.9 × 10^22^, and the new algorithm in time proportional to 9000 + 11000 + 5 · *M*(38, 5) = 4.3 × 10^6^.

### Experiments

We now show the results of the new algorithm applied to the HPO [7]. In our previous work we implemented the Phenomizer as a system for experts in the differential diagnosis in medical genetics; the Phenomizer can be queried with a set of HPO terms to get a ranked list of candidate diseases most similar to the query based on *P*-values derived from Resnik similarity scores, Eq. (3) [17]. However, for the Phenomizer we used Monte Carlo sampling to approximate the score distribution and we will investigate now the difference in using the exact *P*-value compared to sampling.

**Figure 4:**
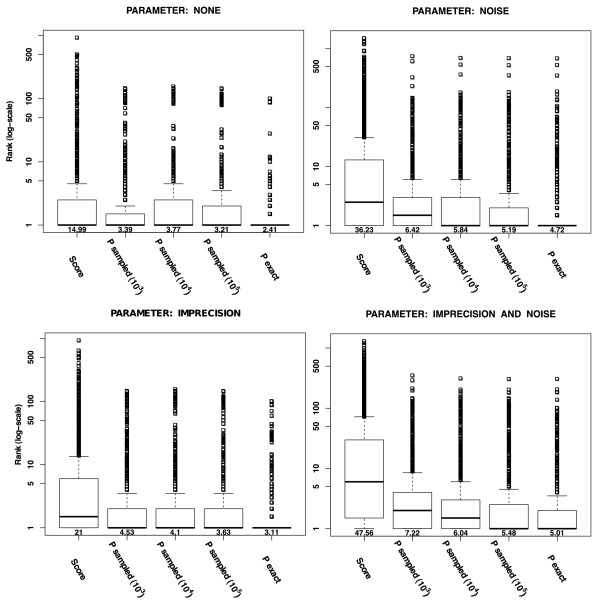
**Impact of Exact *P*-value Computation for Clinical Diagnostics with the HPO**. Simulations for Clinical Diagnostics using the HPO. Patient (phenotype) data was simulated and queried against the complete database of all 4992 annotated diseases. The best result is obtained if the original disease is assigned the rank one (y-axis) by the search algorithm. Different approaches are compared (x-axis). Data were generated without error NONE and with NOISE (top row, left and right) and with IMPRECISION and both IMPRECISION and NOISE (bottom row, left and right) as explained in the Methods section. The mean rank is shown below each boxplot.

As we are interested in ranking diseases for differential diagnosis we will take a similar simulation approach as in [17] and generate sets of artificial patients for which we know the OMIM disease, see Methods. In Figure 4 the results are shown for the investigated scenarios NONE, NOISE, IMPRECISION, and NOISE + IMPRECISION. We compared the ranking of patients with the similarity score alone, sampling based *P*-values (10^3 ^- 10^5 ^repetitions, the latter used in the Phenomizer), and exact computation using the algorithm in this work. In all cases, using the exact *P*-value computation significantly outperforms the four alternative ranking methods (Mann-Whitney *P*-value < 0.001) and ranks the true disease on rank one most of the time. The improvement for the exact score distribution computation is due to the fine-grained resolution especially for small *P*-values, where sampling is often underrepresented, but which are important for selecting the best rank (see Additional File 1).

We then investigated the runtime for different *q *values as compared to using the naive algorithm and Monte Carlo sampling (Table 1). For that purpose we selected four diseases with a different number of annotated HPO terms, and therefore different size of $T^{IMPL}$, and show the runtime of the three approaches in milliseconds. The naive algorithm cannot be utilized for *q *> 2. The exact *P*-value computation is faster than random sampling with 10^5 ^repetitions for *q *= 2,3 and for the disease with only 17 terms in $T^{IMPL}$ independent of the analyzed *q*. Starting from *q *= 4 the sampling based approach is faster for large $\left|T^{IMPL}\right|$ because of the huge size of the score distribution to be computed, but even for *q *= 5 the complete score distribution can be computed in under 4 seconds for diseases with many annotations. Note again that the average size of $T^{IMPL}$ is 38 in the HPO.

**Table 1:** Runtime in milliseconds averaged over 20 runs comparing the naive, exact, and sampled distribution computation for q = 2,3,4, and 5

Runtime Analysis with the HPO						
				***runtime in milliseconds***		
**OMIM ID**	|T|	$\left|T^{IMPL}\right|$	|U|	**naive**	**exact**	sampled*

*q *= 2						
264300	5	17	16	3779	4	50
613124	7	36	36	3794	6	53
113450	12	80	72	3789	6	65
129500	20	66	61	3702	15	89

*q *= 3						
264300	5	17	16	~ 1.2 · 10^7^	4	49
613124	7	36	36	~ 1.2 · 10^7^	6	53
113450	12	80	72	~ 1.2 · 10^7^	19	66
129500	20	66	61	~ 1.2 · 10^7^	15	79

*q *= 4						
264300	5	17	16	-	5	46
613124	7	36	36	-	20	55
113450	12	80	72	-	250	65
129500	20	66	61	-	135	77

*q *= 5						
264300	5	17	16	-	7	48
613124	7	36	36	-	141	54
113450	12	80	72	-	3896	63
129500	20	66	61	-	1776	79

Four OMIM diseases with a varying number of annotated HPO terms (|T|) were used; 264300: 17-*β *Hydroxysteroid Dehydrogenase III deficiency, 613124: Hydrops fetalis, nonimmune, with gracile bones and dysmorphic features, 113450: Brachydactyly-distal symphalangism syndrome, 129500: Ectodermal dysplasia 2, hidrotic. Entries denoted "-" were terminated after four hours. *Sampling with 10^5 ^repetitions.

## Discussion

In this work we have tackled the unstudied problem of computing the score distribution for similarity searches with ontologies. We have devised an efficient preprocessing of the underlying DAG of the ontology that reduces the complexity for similarity measures based on Resnik's popular definition of similarity [19]. We have introduced a new algorithm based on multiset enumeration, which can be applied to score distribution computation for Eq. (3) as well as variants based on maximum similarity Eq. (4). In experiments with the HPO, as well as in theory, we show that the new algorithm is much faster than exhaustive enumeration of the score distribution or resampling approaches and that it is applicable to current ontologies.

The algorithm we describe here can be used as a component of a procedure to find the best hit in a database, i.e., we need to calculate the score for each entry in the database and rank the results according to *P*-value. This allows users to enter a list of characteristics or features in order to identify objects whose characteristics best match the query using semantic similarity. We have implemented our algorithm in the setting of medical diagnostics, where the features are the signs and symptoms of diseases and the domain objects are diseases. We have previously shown that this kind of search is useful for medical differential diagnosis [17].

Summarizing all nodes that have the same target set similarity score makes use of the fact that the pairwise similarity defined by Resnik only considers the common ancestors of the relevant terms (Lemma 1). Extending the proposed algorithm for other popular semantic similarity measures based on the information content of a node, like Jiang and Conrath or Lin [20,21], or the symmetric definition of Eq. (3) [12], has not been considered here as definition of pairwise similarity additionally incorporates the information content of the nodes in the query. Therefore, additional steps are necessary which render the computations more complicated. Although this can be considered a limitation of the current approach, we believe the methodology introduced here will prove useful for other measures as well. For example the term overlap similarity measure [22], comparably, only considers common ancestors of query and target set terms, thus an algorithm with similar complexity appears possible from the results presented in this paper. One of the reasons why the *P*-value based rankings outperform the rankings based on scores is that the former account for the annotation bias as observed by Wang et al. [23]. The best-match average semantic similarity measures based on Resnik, like Eq. (3), were shown to have a strong bias. The annotation bias is a further argument to use *P*-values instead of the similarity scores alone.

In the mentioned study by Wang et al. [23], the authors consider the comparison of two proteins via their annotated GO terms, instead of considering any possible subset of the ontology terms as query as in our search setup. Their approach is to compensate for the annotation bias by simulating the distribution of pairwise similarity scores for all annotated ontology term sets and normalizing using a power transformation. Similarly to our experiments, their method might improve when the exact score distribution is computed using our algorithm.

In a practical implementation of our algorithm, the *P*-values could be precomputed for each entry in the database (such as all the diseases in OMIM or each protein in the human proteome). For small *q*, the *P*-values could be calculated dynamically. This might be useful if users are allowed to filter out portions of the database from the search based on some predefined groups (for instance, in genetics, the differential diagnosis might be restricted to diseases showing a certain mode of inheritance).

Due to its simple structure the new algorithm could be parallelized to run with several threads with close to linear speedup, by keeping the scores in different hash structures for each thread and merging all hashes at the end to get the complete distribution. Also, as often only the *P*-value is of interest, a branch and bound formulation of the new algorithm might lead to a significant speedup in practice.

## Conclusions

The algorithmic improvement reported here might prove useful for *P*-value computation of other semantic similarity measures that are based on the information content of a node as introduced by Resnik [12]. However, when the similarity score includes more dependencies the size of the complete score distribution may increase significantly. Further algorithmic development will be necessary to increase the class of similarity measures for which *P*-values can be computed efficiently.

We believe that our methods would be applicable to other applications in which users search for domain objects that best exemplify a set of desired attributes and that they can be used to improve bioinformatic methods that use the semantic similarity scores alone. For that purpose we implemented a software in Java that computes exact score distributions for both similarity measures discussed here. The software works with any ontology available in OBO format and is available for non-commercial and academic usage under: https://compbio.charite.de/svn/hpo/trunk/src/tools/significance/

## Appendix A

In this Appendix, we will prove Theorem 1 for arbitrary *q*. In the following text, we will outline the approach of the proof and introduce a few new definitions. We can calculate the *P*-values, Eq. (6), by computing the frequency *F*_*i *_of each score *S*_*i *_in the score distribution, i.e., by calculating the number of queries that result in score *S*_*i *_for each possible score. We will consider all query sets Q that result in score *S*, denoted as $Q_{S}$ later in Eq. (15). These initial query sets consist of the nodes from the Ontology DAG G=(V,E). Subsequently, we will substitute sets of nodes Q by multisets $M^{q}\left(Q\right)$ over their target set similarity scores in Eq. (16). This is the important switch that establishes the independence of the number of nodes in the graph by only considering their target set similarity scores. At this step, changing from sets to multisets is necessary, because the same target set similarity score may occur more than once given nodes in a single Q. However, the induced multisets from all sets in $Q_{S}$ are themselves not unique and therefore we will use the multiset frequency, Eq. (12), over the set of unique multisets $M_{S}^{q}$ given $Q_{S}$ to compute the desired quantity *F *in the proof.

We are interested in the set $Q_{S}$ of all sets {*n*_1_,..., *n*_*q*_} of nodes {*n*_1_,..., *n*_*q*_} ⊆ *V*, which result in the same average score *S*. That is, $Q_{S}$ is the set of all queries of size *q *that result in the same average score *S*:

(15) $$Q_{S}=\left\{\left\{n_{1},\ldots ,n_{q}\right\}|\left\{n_{1},\ldots ,n_{q}\right\}\subseteq V,sim^{avg}\left(\left\{n_{1},\ldots ,n_{q}\right\},T\right)=S\right\}.$$

The core message of Theorem 1 is that we can define a multiset *M*^*q *^over the target set similarity scores *s *whose frequency can be used to compute the frequency *F *of scores *S *in the score distribution. A necessary first step therefore is to express a query set $Q=\left\{n_{1},\ldots ,n_{q}\right\}\subseteq V$ as a multiset $M^{q}\left(Q\right)$:

(16) $$M^{q}\left(Q\right)=\left\{\left(s_{1},m_{1}\right),\ldots ,\left(s_{o},m_{o}\right)|s_{i}\in U_{Q},m_{i}=m_{s_{i}}^{Q}\right\},$$

where

(17) $$U_{Q}=\left\{s_{i}|n_{i}\in Q,sim\left(n_{i},T\right)=s_{i}\right\}$$

and

(18) $$m_{s_{i}}^{Q}=|\left\{n_{i}|n_{i}\in Q,sim\left(n_{i},T\right)=s_{i}\right\}|.$$

The underlying set UQ for a multiset $M^{q}\left(Q\right)$ consists of all existing distinct target set similarity scores *s*_*i *_of the nodes in Q, Eq. (17), and their multiplicity is the number of nodes in Q that share the same score *s*_*i*_, Eq. (18).

Now that we know how to create a multiset of target set similarity scores from any given set of nodes in *V*, we need another variable $M_{S}^{q}$ to represent all distinct multisets that can be generated using Eq. (16) from the set $Q_{S}$. The set of distinct multisets $M_{S}^{q}$ generated for a given $Q_{S}$ is defined as:

(19) $$M_{S}^{q}=\left\{M^{q}\left(Q\right)|Q\in Q_{S}\right\}.$$

We can now state the proof of Theorem 1 as follows.

*Proof*.

(20) $$F=|Q_{S}|$$

(21) $$=\underset{M^{q}\in M_{S}^{q}}{\sum }\underset{\left(s,m\right)\in M^{q}}{\prod }\left(\begin{array}{c} \left|N_{s}\right|\\ m \end{array}\right)$$

(22) $$=\underset{M^{q}\in M_{S}^{q}}{\sum }freq\left(M^{q}\right)$$

(23) $$=\underset{M^{q}\in M_{all}^{q},\;sim^{avg}\left(M^{q}\right)=S}{\sum }freq\left(M^{q}\right)$$

Eq. (20) merely restates the definition of the Frequency *F *given by Eq. (15), namely the number of all queries Q⊆V that result in *sim*^*avg *^= *S*. Note that Eq. (15) is representing the number of such queries in terms of sets of nodes of the ontology. Eq. (21) switches the representation from nodes in *V *to multisets $M_{S}^{q}$ over the similarity scores of nodes in *V *using Eq. (19) and the definition of multiset frequency given in Eq. (12). Eq. (22) follows directly from the definition of the multiset frequency in Eq. (12). The equality between Eq. (22) and (23) is a direct consequence of Eq. (15) and (19).

## Authors' contributions

MHS, SK, and PNR planned the research work. MHS, and SB designed the algorithm. MHS, SK, and SB implemented the software and made the analysis. All authors wrote the paper and approved the final manuscript.

## Supplementary Material

Additional file 1 **Additional File **1**contains some additional plots showing the differences in ranking by exact and sampled *P*-values for Clinical Diagnostics with the HPO**. Click here for file
