# Supplementary material for: Exact score distribution computation for ontological similarity searches
Source: BMC Bioinformatics. 2011 Nov 12;12:441. doi: 10.1186/1471-2105-12-441 (PMC3240574; doi:10.1186/1471-2105-12-441)
Supplement: Additional file 1 — contains some additional plots showing the differences in ranking by exact and sampled P-values for Clinical Diagnostics with the HPO. [file 1471-2105-12-441-S1.PDF]

Supplemental Material
Exact Score Distribution Computation for Ontological Similarity Searches

Marcel H. Schulz [1,2,†], Sebastian Köhler [3,4], Sebastian Bauer [3], Peter N. Robinson [1,4,†]

[1]Max Planck Institute for Molecular Genetics, Ihnestr. 73, 14195 Berlin, Germany
[2]Ray and Stephanie Lane Center for Computational Biology, Carnegie Mellon University, 5000 Forbes Avenue, Pittsburgh, 15213 Pennsylvania, United States
[3]Institute for Medical Genetics, Charité-Universitätsmedizin Berlin, Augustenburger Platz 1, 13353 Berlin, Germany
[4]Berlin-Brandenburg Center for Regenerative Therapies (BCRT), Charité-Universitätsmedizin Berlin, Berlin, Germany
[†] corresponding authors

Detailed Rankings for Sampled and Exact P-values

Here, we show the effect of the exact P-value computation of the indiviudal ranks in a direct comparison to the sampled P-values as obtained by Monte-Carlo sampling. Clearly, the more sampling repetitions are undertaken the better the rank as compared to the exact P-value based ranking, see Figure 1. However, especially the best (i.e., smallest) ranks depend on small P-values, the tail of the score distribution, which is often underrepresented in the sampling approach.

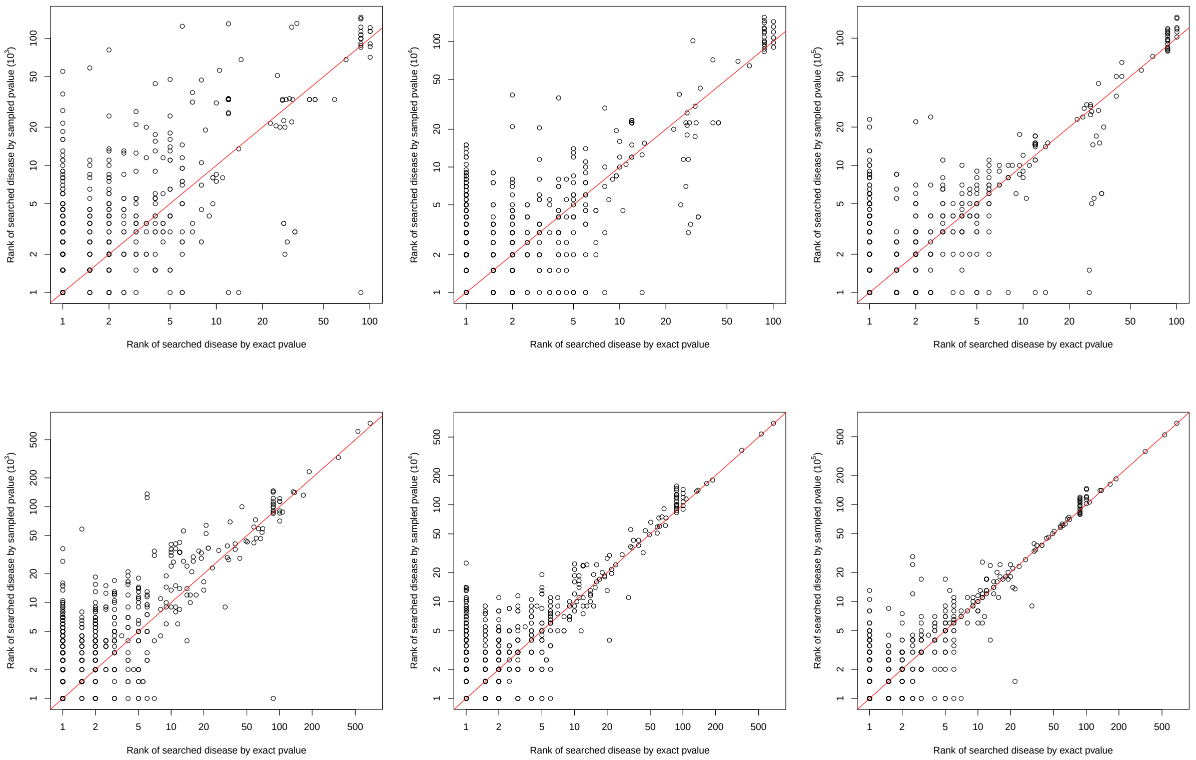

Supplemental Figure 1: Simulations for Clinical Diagnostics using the HPO. Patient phenotype data were generated by perturbing with IMPRECISION (top row) or NOISE (bottom row) as explained in the Methods section of the main manuscript. The simulated patients were queried against the complete database of all 4992 annotated diseases. The result is optimal if the original disease is placed at the first rank by the search procedure. The scatterplots compare the rank achieved with sampling P-values (y-axis) and exact P-values (x-axis). Sampling was done with $10^3 - 10^5$ repetitions (from left to right).
